# High Glucose Attenuates Cardioprotective Effects of Glucagon-Like Peptide-1 Through Induction of Mitochondria Dysfunction *via* Inhibition of β-Arrestin-Signaling

**DOI:** 10.3389/fphys.2021.648399

**Published:** 2021-05-13

**Authors:** Xietian Pan, Chengxiang Li, Haokao Gao

**Affiliations:** ^1^Department of Cardiology, People’s Liberation Army General Hospital, Beijing, China; ^2^Department of Cardiology, Xijing Hospital, Air Force Medical University, Xi’an, China

**Keywords:** GLP-1, β-arrestin, mitochondria dysfunction, diabetic cardiomyocyte, PI3K/Akt

## Abstract

An increased vulnerability has been detected after ischemia/reperfusion injury in cardiomyocytes in diabetic patients. Glucagon-like peptide-1 (GLP-1) has been proven to have a notable cardioprotective effect in cardiomyocytes. However, in diabetic patients, the cardioprotective effects of GLP-1 are compromised, which is called GLP-1 resistance. β-arrestin is one of the two main downstream effectors of GLP-1 and β-arrestin signaling pathway exerts cardioprotective effects upon activation of GLP-1R. Our hypothesis is that the increased vulnerability of cardiomyocytes in diabetic patients is partly due to disruption of the β-arrestin signaling pathway. To test this, we analyzed cardiomyocyte viability and survival in high glucose and normal glucose condition after hypoxia/reoxygenation injury *in vitro*, additional GLP-1 was used to determine whether β-arrestin signaling pathway was involved. We also investigated the role of mitochondrial dysfunction in GLP-1 resistance. Our results showed that cardioprotective effects of GLP-1 were reduced in high glucose cultured H9C2 cells compared to normal glucose cultured H9C2, verifying the existence of GLP-1 resistance in high glucose cultured H9C2 cells. Further study suggested that β-arrestin plays a key role in GLP-1 resistance: β-arrestin expression is notably downregulated in high glucose condition and cardioprotective effects of GLP-1 can be diminished by downregulation of β-arrestin in normal glucose condition while upregulation of β-arrestin can restore cardioprotective effects of GLP-1 in high glucose condition. Then we explore how β-arrestin affects the cardioprotective effects of GLP-1 and found that β-arrestin exerts cardioprotective effects by improving mitochondria quality control via the PI3K/Akt signaling pathway. Thus, our study found out a new mechanism of GLP-1 resistance of cardiomyocytes in high glucose conditions that impaired β-arrestin expression, caused mitochondria dysfunction and eventually cell death. Our study provided a new perspective in treating myocardial ischemia/reperfusion injury in diabetic patients.

## Introduction

Evidence showed that diabetic patients are two to three times more likely to have cardiovascular disease than non-diabetic patients (Danaei et al., 2006). Cardiovascular complications are the major causes of morbidity and mortality in diabetic patients, among which ischemic heart disease is the leading cause of death (Emerging Risk Factors Collaboration et al., 2010). It has been proven that cardiomyocytes exhibit increased vulnerability in diabetic patients, and myocardial infarction causes more cell death in diabetic patients than in non-diabetic patients (Lopaschuk et al., 2010; Smith et al., 2016). Modern medicine restores myocardial perfusion through either percutaneous coronary intervention or thrombolytic therapy, and both of these have brought about a new problem that aggravates cardiomyocyte damage: ischemia/reperfusion injury (Kolwicz et al., 2013). It has been shown that diabetic patients suffered severer damage from ischemia/reperfusion injury (Marfella et al., 2002; Palee et al., 2020). The mechanisms underlying the increased vulnerability of cardiomyocytes in diabetic patients remained unknown.

Secreted from intestinal L cells, GLP-1 exerts insulinotropic effect upon binding to its receptor in islet B cell and serves as a clinical used anti-diabetic drug (Baggio and Drucker, 2007). GLP-1 also exerts cardioprotective effects through binding to its receptor in cardiomyocytes (Croston et al., 2014; Helmstadter et al., 2020). Research found that GLP-1 improves cardiac function, decreases infarct size after myocardial ischemia/reperfusion injury, thus making GLP-1 a promising drug that can be used in dealing with cardiomyocyte injury of diabetic patients (Tao et al., 2018). However, studies have found that there exists GLP-1 resistance in diabetic conditions. Thus, uncovering the mechanisms underlying GLP-1 resistance is of crucial importance.

Being one of the class B G-protein-coupled receptor family, GLP-1R exerts its physiological function mainly through two pathways: GPCR dependent pathway and GPCR-independent pathway (Noyan-Ashraf et al., 2009). The latter is also called the β-arrestin signaling pathway, which is proven to exerts numerous functions including cell survival (Thomsen et al., 2016). This raises questions about whether it is involved in GLP-1 resistance. Another vital element when discussing cardiomyocyte viability is mitochondria. Mitochondria quality control is crucial in maintaining hemostasis in cardiomyocytes (Mughal et al., 2018; Silverblatt et al., 2019). Mitochondria dysfunction may eventually result in apoptosis or necrosis (Bock and Tait, 2020). The role of mitochondria dysfunction in GLP-1 resistance is worthy of exploring. In this study, we aimed to reveal the underlying mechanism of GLP-1 resistance in diabetic cardiomyocytes, explore the role of β-arrestin and mitochondria dysfunction in GLP-1 resistance and shed light on future therapy.

## Materials and Methods

### Cell Culture and Establishment of Hypoxia/Reoxygenation Injury Model

Cardiac myoblast cell lines H9C2 were obtained from the Cell Bank of the Chinese Academy of Sciences (Shanghai, China). All commercially available kits and agents used in the present study are listed in Table 1. Cells were cultured in DEME containing 10% neonatal bovine serum and 1% penicillin/streptomycin and incubated in a humidified chamber with 95% ambient air and 5% CO_2_ at 37°C. Cells were grown at 1.2 × 10^5^ in 6-well plates for 21 days, changing the media each 48 h without passaging the cells. High glucose (HG) and normal glucose (NG) condition were defined as 33 and 5 mM, respectively in this study. Hypoxia/Reoxygenation (H/R) injury model of H9C2 cells was obtained by exposure to hypoxia (95% N_2_, 5% CO_2_) in an anaerobic system (Thermo Forma) at 37°C for 6 h followed by reoxygenation in normoxia (95% ambient air, 5% CO_2_) for 10 h as previously reported (Pan et al., 2019). In the control group, H9C2 cells were maintained at normoxia for equivalent periods. GLP-1R agonist Exentin-4 (Ex-4) was purchased from Sigma and for Ex-4 pre-treated group 50 nM Ex-4 was added 1 h before H/R.

**TABLE 1:** Primer sequences.

**Gene symbol**	**Sequence**
Bcl-2	5′-GTCGCTACCGTCGTGACTTC
	3′-CAGACATGCACCTACCCAGC
BAX	5′-TGAAGACAGGGGCCTTTTTG
	3′-AATTCGCCGGAGACACTCG
Drp1	5′-TCCCTAAACTCCATGATGCCATA
	3′-CCACAGGCATCAGCAAAGTC
Mff	5′-ATGCCAGTGTGATAATGCAAGT
	3′-CTCGGCTCTCTTCGCTTTG
Atg5	5′-TGTGCTTCGAGATGTGTGGTT
	3′-ACCAACGTCAAATAGCTGACTC
Beclin-1	5′-CAGGAGAGACCCAGGAGGAA
	3′-GCTGTTGGCACTTTCTGTGG
β-arrestin	5′-AAGGGACACGAGTGTTCAAGA
	3′-CCCGCTTTCCCAGGTAGAC

### siRNA and Adenovirus Transfection

For siRNA transfection, β-arrestin siRNA and scrambled siRNA were purchased from Santa Cruz Biotechnology (Santa Cruz, United States). H9C2 cells were transfected with the siRNAs using Lipofectamine2000 Reagent (Life Invitrogen, United States) according to the manufacturer’s instruction.

Recombinant β-arrestin-expressing adenoviruses were constructed by Genechem (Shanghai, China). H9C2 cells were transfected with β-arrestin-expressing adenoviruses and adenoviruses containing empty plasmids (control) according to the manufacturer’s instruction.

### TUNEL Staining and Caspase-3 Activity

TUNEL staining was performed according to the manufacturer’s instructions (MEBSTAIN Apoptosis TUNEL kit, Takara). In brief, H9C2 cells were incubated with the terminal deoxynucleotidyl transferase (TdT) enzyme and 2′-deoxyuridine 5′-triphosphate (dUTP) at 37°C for 1 h. Then, the nuclei were stained with 4′,6-diamino-2-phenylindole (DAPI) for 5 min. Digital photographs were taken at high magnification (×400) using fluorescent microscopy (Olympus). Cells with stained nuclei were defined as TUNEL positive. Apoptosis index (AI) is defined as apoptotic cell number in 100 cells averagely. Each AI was accessed in 20 randomly selected fields.

Caspase-3 activity was measured using a caspase-3 assay kit (Clontech, Mountain View, CA) according to the manufacturer’s instructions.

### ROS Measurement

ROS level was measured with the dye 2′,7′-dichlorofluorescein diacetate (DCFH-DA) according to the manufacturer’s instructions. The resulting fluorescence was quantified using ImageJ software and expressed as mean fluorescence intensity.

### Cell Counting Kit-8 Assay

The CCK-8 assay was conducted according to the manufacturer’s instructions. In brief, cells in the logarithmic growth phase were trypsinized and inoculated into 96-well plates with 100 μl of cell suspension per well. After 24 h, each well was supplied with 10 μl of cell counting kit-8 (CCK8) liquor (Hubei Bios Biotechnology Co., Ltd.). After incubation for 1 h, a microplate reader was then used to measure the absorbance [optical density (OD) value] of the 96-well plates at a 450-nm wavelength. OD values of the cells were measured to evaluate cell viability.

### Real Time Quantitative PCR

Total RNA was isolated from H9C2 cells using TRIzol reagent (Invitrogen, United States) according to the manufacturer’s instructions. Real-time quantitative PCR was conducted following standard methods. Primer sequences are provided in Table 2.

**TABLE 2:** Catalog numbers of commercially available kits and agents.

**Product name**	**Company**	**Catalog no.**
Exendin-4	Abcam, United Kingdom	ab120214
Lipofectamine2000 Reagent	Life Invitrogen, United States	11668500
Wortmannin	MCE, United States	HY-10197
IGF-1	MCE, United States	HY-P7070
TRIzol reagent	Invitrogen, United States	15596018
MEBSTAIN Apoptosis TUNEL kit	Takara, Japan	MK500
DAPI	Abcam, United Kingdom	ab228549
Caspase-3 assay kit	Clontech, CA	630217
ROS assay kit	Abcam, United Kingdom	ab113851
CCK-8 assay kit	Sigma, United States	96992-100TESTS-F

### Statistical Analysis

Results are represented as mean ± SD. Statistical analyses were performed by one-way analysis of variance (AVONA), and a Student-Newman-Keuls multiple comparison test was performed to identify differences between groups. A *P*-value of less than 0.05 was considered statistically significant.

## Results

### High Glucose Condition Attenuates Cardioprotective Effects of Glucagon-Like Peptide-1

**FIGURE 1:**
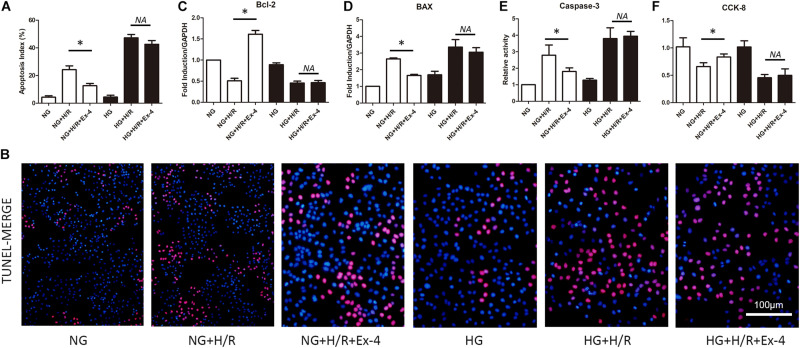
High glucose condition attenuates cardioprotective effects of glucagon-like peptide-1. **(A,B)** TUNEL staining was conducted and apoptosis index (%) was calculated. **(C,D)** Anti-apoptotic Bcl-2 and pro-apoptotic BAX were measured using qRT-PCR. **(E)** Caspase-3 activity was measured using caspase-3 activity kit. **(F)** Cell viability was measured via CCK-8 assay. *P < 0.05.

As we introduced above, diabetic patients exhibited vulnerable cardiomyocytes when experiencing stress-like ischemia, and GLP-1 failed to exert intact cardioprotective effects in diabetic cardiomyocytes. Here we first investigated whether a high glucose condition could impair the cardioprotective effects of GLP-1 *in vitro*. As shown in Figures 1A,B, H/R injury caused more cell death in high glucose cultured H9C2 cells compared to normal glucose cultured H9C2 cells. Additional GLP-1R agonist Exentin-4 exerted anti-apoptosis effects in normal glucose but not in high glucose cultured H9C2 cells. On a molecular level, we obtained similar results that H/R injury in the HG group increased expression of pro-apoptotic BAX (Figure 1D) and caspase-3 activity (Figure 1E) while reduced cell viability (Figure 1F) and expression of anti-apoptotic Bcl-2 (Figure 1C) compared to NG group. Exendin-4 could effectively reverse the pro-apoptotic effect of H/R injury in the NG group by inhibition of BAX expression and caspase-3 activity and induction of Bcl-2 expression, while in the HG group such effects vanished.

### Increased Susceptibility of Mitochondrial Dysfunction Attenuated Cardioprotective Effects of GLP-1 in High Glucose Cultured H9C2 Cells

**FIGURE 2:**
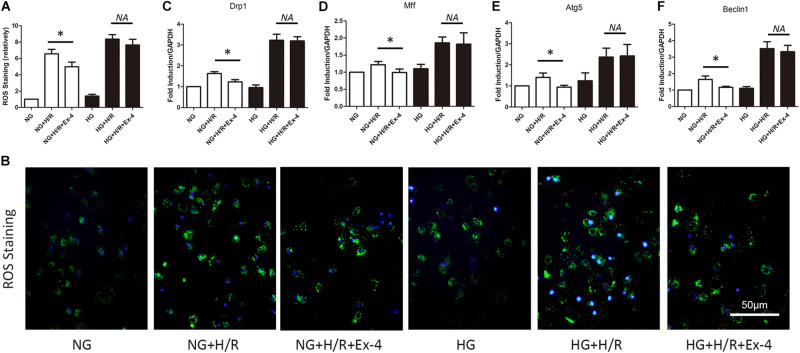
Increased susceptibility of mitochondrial dysfunction attenuated cardioprotective effects of GLP-1 in high glucose cultured H9C2 cells. **(A,B)** Measurement of mitochondrial function using ROS staining. **(C,D)** Detection of mitochondrial fission level by qRT-PCR measurement of Drp1 and Mff. **(E,F)** Detection of mitophagy level by qRT-PCR measurement of Atg5 and Beclin1. *P < 0.05.

It is well-known that mitochondria play a crucial role in cell survival. Dysfunction in mitochondrial quality control eventually caused cell death either by apoptosis or necrosis. Here, we explore whether decreased cardioprotective effects of GLP-1 were associated with mitochondrial dysfunction in high glucose conditions. As was shown in Figures 2A,B, Exendin-4 could restore the increase of ROS level in normal glucose cultured H9C2 cells after H/R injury but not in high glucose conditions. Similar results were obtained when detecting biomarkers of mitochondrial fission dynamin-related protein 1 (Drp1), mitochondrial fission factor (Mff) (Figures 2C,D), and biomarkers of mitophagy autophagy-related gene 5 (Atg5) and Beclin1 (Figures 2E,F).

### β-Arrestin Overexpression Restored Cardioprotective Effects of GLP-1 in High Glucose Cultured H9C2 Cells

**FIGURE 3:**
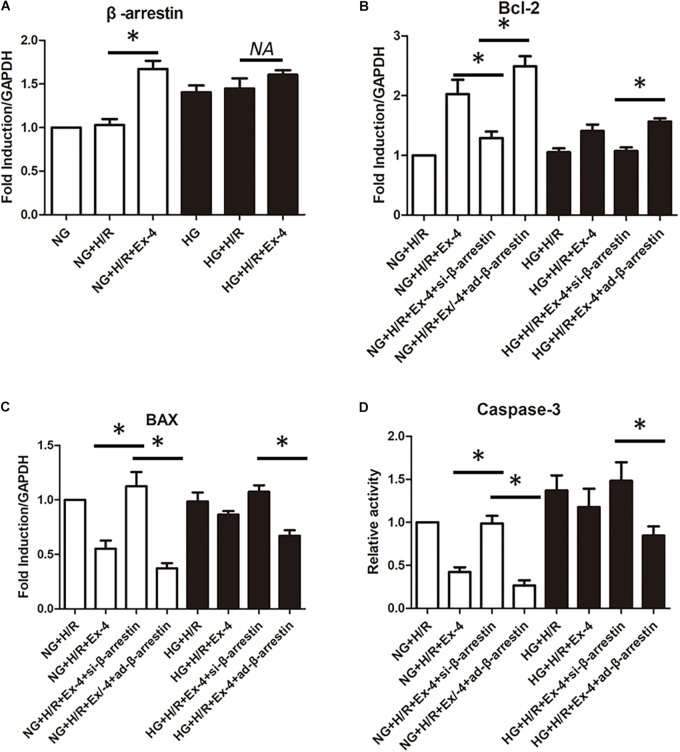
β-arrestin overexpression restored cardioprotective effects of GLP-1 in high glucose cultured H9C2 cells. **(A)** qRT-PCR measurement of β-arrestin transcription. **(B–D)** Exploring the changes in cardioprotective effects of Exendin-4 while utilizing β-arrestin siRNA and adenovirus transfection. *P < 0.05.

GLP-1 exerts its physiological function mainly through the GPCR-dependent signaling pathway and GPCR-independent signaling pathway. The latter is also called the β-arrestin signaling pathway. It has been proven that the β-arrestin signaling pathway is associated with cell survival, which brings us to question whether the β-arrestin signaling pathway is involved in GLP-1 resistance in high glucose cultured H9C2 cells. Here, we first investigate the change of β-arrestin expression in each group and found that high glucose condition itself could decrease β-arresting expression in H9C2 cells, and the addition of GLP-1 could hardly upregulate the expression of β-arrestin in high glucose cultured H9C2 cells (Figure 3A). By upregulating and downregulating β-arrestin expression by β-arrestin si-RNA and β-arrestin adenovirus, respectively, we further explored the role of β-arrestin in GLP-1 resistance in high glucose cultured H9C2 cells. As was shown in Figures 3B–D, the cardioprotective effects of Exendin-4 can be diminished by downregulation of β-arrestin in normal glucose conditions, while upregulation of β-arrestin can restore cardioprotective effects of Exendin-4 in high glucose condition.

### High Glucose Attenuates Cardioprotective Effects of GLP-1 Through Induction of Mitochondrial Dysfunction *via* Inhibition of β-Arrestin-Signaling

**FIGURE 4:**
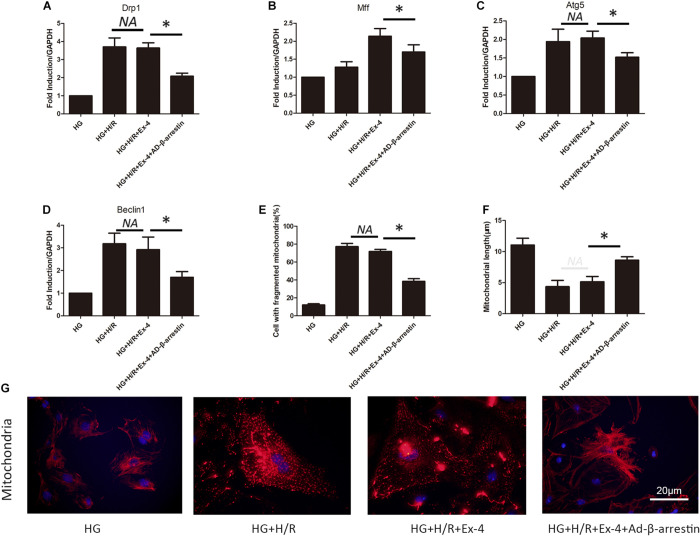
High glucose attenuates cardioprotective effects of GLP-1 through induction of mitochondrial dysfunction via inhibition of β-arrestin-signaling. **(A,B)** Mitochondrial fission level measured by qRT-PCR analysis of Drp1 and Mff. **(C,D)** Mitophagy level measured by qRT-PCR analysis of Atg5 and Beclin1. **(E–G)** Detection of mitochondria morphology using mitochondria staining. *P < 0.05.

A previous study revealed the relationship between attenuated cardioprotective effects of GLP-1 in high glucose cultured H9C2 cells and β-arrestin. Mitochondrial dysfunction was also found to be involved in GLP-1 resistance in high glucose cultured H9C2 cells. Here, we further investigated whether high glucose attenuates the cardioprotective effects of GLP-1 through induction of mitochondrial dysfunction via inhibition of β-arrestin-signaling. As shown in Figures 4A,B, the upregulation of β-arrestin could effectively attenuate mitochondrial fission. Similar results can be found in mitophagy biomarkers Atg5 and Beclin1 (Figures 4C,D). Indicating that β-arrestin-signaling exerts cardioprotective effects via inhibition of mitochondrial dysfunction. Detailed mitochondria staining was also detected and it was shown that upregulation of β-arrestin increased the number of intact mitochondria (Figures 4E–G).

### Upregulation of β-Arrestin Attenuates Mitochondrial Dysfunction *via* the PI3K/Akt Signaling Pathway

**FIGURE 5:**
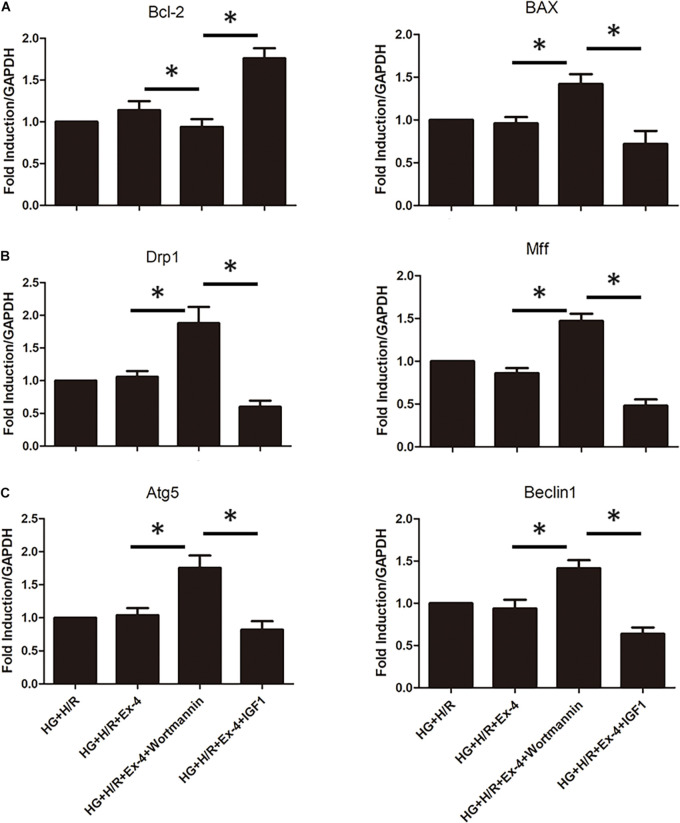
Upregulation of β-arrestin attenuates mitochondrial dysfunction via PI3K/Akt signaling pathway. **(A)** qRT-PCR analysis of apoptotic biomarkers Bcl-2 and BAX. **(B)** qRT-PCR analysis of mitochondrial fission biomarkers Drp1 and Mff. **(C)** qRT-PCR analysis of mitophagy biomarkers Atg5 and Beclin1. *P < 0.05.

In a previous study, we associated GLP-1 resistance with β-arrestin signaling and mitochondrial dysfunction. It was well-known that there exist several signaling pathways downstream of β-arrestin, including the cell survival pathway of PI3K-Akt. Here we investigate whether β-arrestin regulates mitochondrial dysfunction via PI3K-Akt signaling pathway. We used wortmannin as an inhibitor of the PI3K/Akt pathway and IGF1 as an activator of the PI3K/Akt pathway. As was in Figure 5A, regulation of the PI3K/Akt pathway could notably affect apoptotic biomarkers Bcl-2 and BAX. Similar results could be found in mitochondrial fission and mitophagy (Figures 5B,C). *^∗^P* < 0.05.

## Discussion

In this study, we verified the existence of GLP-1 resistance in high glucose cultured H9C2 cells after H/R injury. We found that the cardioprotective effects of GLP-1 are notably attenuated in high glucose conditions after H/R injury. Further investigation revealed that there existed β-arrestin expression downregulation in high glucose conditions; downregulation of β-arrestin in normal glucose conditions diminished the cardioprotective effects of GLP-1, while upregulation of β-arresting can restore cardioprotective effects of GLP-1 in high glucose condition. We found that disruption of β-arrestin expression in high glucose conditions after H/R injury caused mitochondrial dysfunction through the PI3K/Akt pathway and eventually leading to cell death. To our knowledge, this is the first study to describe the role of β-arrestin and mitochondrial dysfunction in GLP-1 resistance in high glucose cultured cardiomyocytes.

Numerous studies have investigated the effects of GLP-1 in cardiomyocytes and found that GLP-1 can improve cardiomyocyte contractility, induce cell survival signaling, inhibit apoptosis, reduce blood pressure, and inhibit hypertrophy (Thomsen et al., 2016; Weis and Kobilka, 2018). GLP-1 exerts its physiological function mainly through two pathways, of which the β-arrestin signaling pathway mainly exerts cardioprotective effects (Sokos et al., 2006). However, studies have found that in diabetic mice there exists GLP-1 resistance in several cell types such as pancreatic β cells and endothelial cells (Xu et al., 2007; Ceriello et al., 2011). Our study revealed that there exists GLP-1 resistance in high glucose cultured H9C2 cells after H/R injury, and it is by downregulation of β-arrestin expression that high glucose attenuated the cardioprotective effects of GLP-1 after H/R injury.

Mitochondrial function is essential to the physiology and pathology of adult hearts. It has been found that mitochondria shape changed after ischemia and reperfusion injury in cardiomyocytes, which is later called mitochondrial fragmentation (Ashrafian et al., 2007; Gargiulo et al., 2020). Studies also revealed that inhibition of fusion or promotion of fission may induce excessively fragmented mitochondria (Chen et al., 2003; Wu et al., 2017). With the understanding of mitochondria dynamics developed, the concept of mitochondria quality control was introduced. MQC includes mitochondrial fission, mitochondrial fusion, mitophagy, and mitochondrial-dependent cell death (Maneechote et al., 2017; Forini et al., 2019). In brief, excessive mitochondrial fission is an early marker of mitochondrial damage and cardiomyocyte death, and mitochondrial fusion has been observed to be associated with stressed cardiomyocytes and cardiac depression (Seidel et al., 2019). Mitophagy keeps the homeostasis of the mitochondrial network, and either excessive or reduced mitophagy results in an unbalanced mitochondrial network and caused cell death (Son and Lee, 2019). Mitochondria have been proven to play a key role in I/R injury of cardiomyocytes (Silverblatt et al., 2019). Mitochondria dysfunction itself can trigger programmed cell death (Del Re et al., 2019). On the other hand, mitochondria dysfunction causes other pathological conditions, such as calcium overload, oxidative stress, endoplasmic reticulum stress, and immune response, all of which could lead to cell death (Zhang et al., 2016). Thus, it cannot be ignored when discussing attenuated cardioprotective effects of GLP-1 after I/R injury. In our study, we found that there exists mitochondrial dysfunction in high glucose cultured cardiomyocytes after H/R injury. Further study showed that mitochondrial dysfunction in high glucose cultured cardiomyocytes after H/R injury is partly regulated by reduced β-arrestin expression.

In conclusion, the present study revealed part of the underlying mechanism of GLP-1 resistance in diabetic cardiomyocytes from a new perspective. We found that high glucose condition itself can impair the downstream cardioprotective signaling pathway of GLP-1R by inhibition of β-arrestin and upregulation of mitochondrial dysfunction. However, the present study has some certain limitations. The key evidence of the paper is mainly supported by gene expression. It would strengthen the data with more evidence of changes in protein expression. For some technical reasons, we did not use animal models to further verify our hypothesis *in vivo*. We revealed part of the mechanisms of GLP-1 resistance, but we did not come up with a solution. Further studies should be focused on restoring the cardioprotective effects of GLP-1 in diabetic individuals.

## Data Availability Statement

The raw data supporting the conclusions of this article will be made available by the authors, without undue reservation.

## Author Contributions

HG and CL designed the experiments. HG and XP performed the experiments. CL and XP contributed to the data collection, statistical analysis, figures preparation, and wrote the manuscript. All authors contributed to the article and approved the submitted version.

## Conflict of Interest

The authors declare that the research was conducted in the absence of any commercial or financial relationships that could be construed as a potential conflict of interest.

## References

[B1] Ashrafian H. Frenneaux M. P. Opie L. H. (2007). Metabolic mechanisms in heart failure. *Circulation* 116 434–448. 10.1161/CIRCULATIONAHA.107.702795 17646594

[B2] Baggio L. L. Drucker D. J. (2007). Biology of incretins: GLP-1 and GIP. *Gastroenterology* 132 2131–2157. 10.1053/j.gastro.2007.03.054 17498508

[B3] Bock F. J. Tait S. W. G. (2020). Mitochondria as multifaceted regulators of cell death. *Nat. Rev. Mol. Cell Biol.* 21 85–100. 10.1038/s41580-019-0173-8 31636403

[B4] Ceriello A. Esposito K. Testa R. Bonfigli A. R. Marra M. Giugliano D. (2011). The possible protective role of glucagon-like peptide 1 on endothelium during the meal and evidence for an “endothelial resistance” to glucagon-like peptide 1 in diabetes. *Diabetes Care* 34 697–702. 10.2337/dc10-1949 21273492 PMC3041210

[B5] Chen H. Detmer S. A. Ewald A. J. Griffin E. E. Fraser S. E. Chan D. C. (2003). Mitofusins Mfn1 and Mfn2 coordinately regulate mitochondrial fusion and are essential for embryonic development. *J. Cell. Biol.* 160 189–200. 10.1083/jcb.200211046 12527753 PMC2172648

[B6] Croston T. L. Thapa D. Holden A. A. Tveter K. J. Lewis S. E. Shepherd D. L. (2014). Functional deficiencies of subsarcolemmal mitochondria in the type 2 diabetic human heart. *Am. J. Physiol. Heart Circ. Physiol.* 307 H54–H65. 10.1152/ajpheart.00845.2013 24778174 PMC4080178

[B7] Danaei G. Lawes C. M. Vander Hoorn S. Murray C. J. Ezzati M. (2006). Global and regional mortality from ischaemic heart disease and stroke attributable to higher-than-optimum blood glucose concentration: comparative risk assessment. *Lancet* 368 1651–1659. 10.1016/S0140-6736(06)69700-6 17098083

[B8] Del Re D. P. Amgalan D. Linkermann A. Liu Q. Kitsis R. N. (2019). Fundamental Mechanisms of regulated cell death and implications for heart disease. *Physiol. Rev.* 99 1765–1817. 10.1152/physrev.00022.2018 31364924 PMC6890986

[B9] Emerging Risk Factors Collaboration Sarwar N. Gao P. Seshasai S. R. Gobin R. Kaptoge S. (2010). Diabetes mellitus, fasting blood glucose concentration, and risk of vascular disease: a collaborative meta-analysis of 102 prospective studies. *Lancet* 375 2215–2222. 10.1016/S0140-6736(10)60484-9 20609967 PMC2904878

[B10] Forini F. Nicolini G. Kusmic C. Iervasi G. (2019). Protective effects of euthyroidism restoration on mitochondria function and quality control in cardiac pathophysiology. *Int. J. Mol. Sci.* 20:3377. 10.3390/ijms20143377 31295805 PMC6678270

[B11] Gargiulo P. Marsico F. Renga F. Dell’Aversana S. Esposito I. Marciano C. (2020). The metabolic syndrome in heart failure: insights to specific mechanisms. *Heart Fail. Rev.* 25 1–7. 10.1007/s10741-019-09838-6 31414215

[B12] Helmstadter J. Frenis K. Filippou K. Grill A. Dib M. Kalinovic S. (2020). Endothelial GLP-1 (Glucagon-Like Peptide-1) receptor mediates cardiovascular protection by liraglutide in mice with experimental arterial hypertension. *Arterioscler. Thromb. Vasc. Biol.* 40 145–158. 10.1161/atv.0000615456.97862.30 31747801 PMC6946108

[B13] Kolwicz S. C. Jr. Purohit S. Tian R. (2013). Cardiac metabolism and its interactions with contraction, growth, and survival of cardiomyocytes. *Circ. Res.* 113 603–616. 10.1161/CIRCRESAHA.113.302095 23948585 PMC3845521

[B14] Lopaschuk G. D. Ussher J. R. Folmes C. D. Jaswal J. S. Stanley W. C. (2010). Myocardial fatty acid metabolism in health and disease. *Physiol. Rev.* 90 207–258. 10.1152/physrev.00015.2009 20086077

[B15] Maneechote C. Palee S. Chattipakorn S. C. Chattipakorn N. (2017). Roles of mitochondrial dynamics modulators in cardiac ischaemia/reperfusion injury. *J. Cell. Mol. Med.* 21 2643–2653. 10.1111/jcmm.13330 28941171 PMC5661112

[B16] Marfella R. D’Amico M. Di Filippo C. Piegari E. Nappo F. Esposito K. (2002). Myocardial infarction in diabetic rats: role of hyperglycaemia on infarct size and early expression of hypoxia-inducible factor 1. *Diabetologia* 45 1172–1181. 10.1007/s00125-002-0882-x 12189448

[B17] Mughal W. Martens M. Field J. Chapman D. Huang J. Rattan S. (2018). Myocardin regulates mitochondrial calcium homeostasis and prevents permeability transition. *Cell Death Differ.* 25 1732–1748. 10.1038/s41418-018-0073-z 29511336 PMC6180099

[B18] Noyan-Ashraf M. H. Momen M. A. Ban K. Sadi A. M. Zhou Y. Q. Riazi A. M. (2009). GLP-1R agonist liraglutide activates cytoprotective pathways and improves outcomes after experimental myocardial infarction in mice. *Diabetes* 58 975–983. 10.2337/db08-1193 19151200 PMC2661586

[B19] Palee S. Higgins L. Leech T. Chattipakorn S. C. Chattipakorn N. (2020). Acute metformin treatment provides cardioprotection via improved mitochondrial function in cardiac ischemia / reperfusion injury. *Biomed. Pharmacother.* 130:110604. 10.1016/j.biopha.2020.110604 32777704

[B20] Pan X. Chen J. Wang T. Zhang M. Wang H. Gao H. (2019). Essential role of high glucose-induced overexpression of PKCbeta and PKCdelta In GLP-1 resistance in rodent cardiomyocytes. *Diabetes Metab. Syndr. Obes.* 12 2289–2302. 10.2147/DMSO.S215789 31807042 PMC6839579

[B21] Seidel T. Fiegle D. J. Baur T. J. Ritzer A. Nay S. Heim C. (2019). Glucocorticoids preserve the t-tubular system in ventricular cardiomyocytes by upregulation of autophagic flux. *Basic Res. Cardiol.* 114:47. 10.1007/s00395-019-0758-6 31673803 PMC9380897

[B22] Silverblatt J. A. Ziff O. J. Dancy L. Daniel A. Carter B. Scott P. (2019). Therapies to limit myocardial injury in animal models of myocarditis: a systematic review and meta-analysis. *Basic Res. Cardiol.* 114:48. 10.1007/s00395-019-0754-x 31673885 PMC6823299

[B23] Smith A. D. Crippa A. Woodcock J. Brage S. (2016). Physical activity and incident type 2 diabetes mellitus: a systematic review and dose-response meta-analysis of prospective cohort studies. *Diabetologia* 59 2527–2545. 10.1007/s00125-016-4079-0 27747395 PMC6207340

[B24] Sokos G. G. Nikolaidis L. A. Mankad S. Elahi D. Shannon R. P. (2006). Glucagon-like peptide-1 infusion improves left ventricular ejection fraction and functional status in patients with chronic heart failure. *J. Card. Fail.* 12 694–699. 10.1016/j.cardfail.2006.08.211 17174230

[B25] Son J. M. Lee C. (2019). Mitochondria: multifaceted regulators of aging. *BMB Rep.* 52 13–23. 10.5483/bmbrep.2019.52.1.300 30545443 PMC6386233

[B26] Tao A. Xu X. Kvietys P. Kao R. Martin C. Rui T. (2018). Experimental diabetes mellitus exacerbates ischemia/reperfusion-induced myocardial injury by promoting mitochondrial fission: Role of down-regulation of myocardial Sirt1 and subsequent Akt/Drp1 interaction. *Int. J. Biochem. Cell Biol.* 105 94–103. 10.1016/j.biocel.2018.10.011 30381241

[B27] Thomsen A. R. B. Plouffe B. Cahill T. J. III Shukla A. K. Tarrasch J. T. Dosey A. M. (2016). GPCR-G protein-beta-arrestin super-complex mediates sustained g protein signaling. *Cell* 166 907–919. 10.1016/j.cell.2016.07.004 27499021 PMC5418658

[B28] Weis W. I. Kobilka B. K. (2018). The molecular basis of G protein-coupled receptor activation. *Annu. Rev. Biochem.* 87 897–919. 10.1146/annurev-biochem-060614-033910 29925258 PMC6535337

[B29] Wu P. Yuan X. Li F. Zhang J. Zhu W. Wei M. (2017). Myocardial upregulation of cathepsin D by ischemic heart disease promotes autophagic flux and protects against cardiac remodeling and heart failure. *Circ. Heart Fail.* 10:e004044. 10.1161/CIRCHEARTFAILURE.117.004044 28694354 PMC5535800

[B30] Xu G. Kaneto H. Laybutt D. R. Duvivier-Kali V. F. Trivedi N. Suzuma K. (2007). Downregulation of GLP-1 and GIP receptor expression by hyperglycemia: possible contribution to impaired incretin effects in diabetes. *Diabetes* 56 1551–1558. 10.2337/db06-1033 17360984

[B31] Zhang Z. Liu L. Wu S. Xing D. (2016). Drp1, Mff, Fis1, and MiD51 are coordinated to mediate mitochondrial fission during UV irradiation-induced apoptosis. *FASEB J.* 30 466–476. 10.1096/fj.15-274258 26432782

